# Monitoring sodium content in processed and ultraprocessed foods in Argentina 2022: compliance with National Legislation and Regional Targets

**DOI:** 10.1017/S1368980024001423

**Published:** 2024-10-02

**Authors:** Leila Guarnieri, Luciana Castronuovo, Nadia Flexner, Yahan Yang, Mary R L’Abbe, Victoria Tiscornia

**Affiliations:** 1 Fundación Interamericana del Corazón Argentina, Buenos Aires, Argentina; 2 Department of Nutritional Sciences, University of Toronto, Toronto, ON M5S 1A8, Canada

**Keywords:** Na reduction, Processed foods, Ultraprocessed foods, Public health policies, Argentina, Latin America

## Abstract

**Objective:**

To assess the current Na levels in a variety of processed food groups and categories available in the Argentinean market to monitor compliance with the National Law and to compare the current Na content levels with the updated Pan American Health Organisation (PAHO) regional targets.

**Design:**

Observational cross-sectional study.

**Setting and Participants:**

*Argentina. Data were collected during March 2022 in the city of Buenos Aires in two of the main supermarket chains. We carried out a systematic survey of pre-packaged food products available in the food supply assessing Na content as reported in nutrition information panels.*

**Results:**

We surveyed 3997 food products, and the Na content of 760 and 2511 of them was compared with the maximum levels according to the Argentinean law and the regional targets, respectively. All food categories presented high variability of Na content. More than 90 % of the products included in the National Sodium Reduction Law were found to be compliant. Food groups with high median Na, such as meat and fish condiments, leavening flour and appetisers are not included in the National Law. In turn, comparisons with PAHO regional targets indicated that more than 50 % of the products were found to exceed the regional targets for Na.

**Conclusions:**

This evidence suggests that it is imperative to update the National Sodium Reduction Law based on regional public health standards, adding new food groups and setting more stringent legal targets.

Non-communicable diseases are responsible for more than 80 % of all deaths^(1)^, with CVD being the leading cause of death in almost all countries. Over half of deaths from CVD in the Latin American Region are attributable to high blood pressure, with 20–35 % of adults having hypertension^(2)^. There is clear evidence that excessive consumption of salt/Na adversely affects blood pressure, which alone accounts for an estimated 10·8 million deaths in 2019^(3)^.

A recent systematic review around the world has identified a significant inverse correlation between discretionary salt intake and a country’s per capita gross domestic product (*P* < 0·0001)^(4)^. In this study, food groups, such as bread and bakery products, cereals and grains, meat products, and dairy products, were found to be significant contributors to daily Na intake across multiple countries and continents.

Na reduction policies are cost effective in reducing the global burden of CVD^(5–8)^. Research about economic evaluations of individual- and population-based interventions for primary and secondary prevention of CVD among adults in low- and middle-income countries has found that those targeting reduction in salt intake are very cost effective in these countries, with potential to generate economic gains that can be reinvested to improve health and/or other sectors^(9)^. Asaria *et al.* ^(10)^ assessed the financial costs and health effects of a voluntary reduction in the salt content of processed foods by manufacturers plus a mass media campaign to encourage dietary change in twenty-three selected low- and middle-income countries, including Argentina. They estimated that a 15 % reduction in dietary salt intake in Argentina would save 60 000 lives over the period 2006–2015 at a cost of USA$ 0·14 per capita (equivalent to AR$ 16·7 million for a population of Argentina (38 million in 2005). It is, therefore, considered a priority action for non-communicable disease prevention^(11)^. Currently, the salt/Na consumption in the Latin America Region and in Argentina is almost double the recommendation from the WHO^(12)^. While WHO recommends a population-based daily intake of < 5 g of salt (< 2 g of Na) per day/per adult^(13)^, in Latin America, the combined average estimated daily salt intake from studies with urine samples collected after 24 h was 9·7 g/d^(14)^. In Argentina, objective measurements conducted as part of the 2018 National Risk Factor Survey indicated that hypertension affects 40·6 % of the adult population in the country^(15)^. In terms of salt intake, it was estimated at 7·9 g/d using spot urine samples, and 91·8 % of individuals aged 18 years and older consumed 5 g of salt or more per day in Argentina, and therefore do not meet WHO recommendations^(16)^.

Research conducted in Argentina has already established the feasibility of Na reduction in processed foods in the country^(17)^. A reduction of 3 g of salt in the Argentinean diet has been estimated to decrease the prevalence of CVD by over 20 %, also contributing to reduced mortality rates from heart disease by 19·9 %^(18)^.

Argentina was the first country in Latin America to introduce a national legislation (Act 26 905) limiting salt levels across a broad range of food groups (meat and meat products; farinaceous; and soups, dressing and canned soups) and other two main measures including education campaigns for the general population and a restaurant strategy (low-Na menus and restriction of saltshakers). The law entered into force in December 2014, a year after its enactment, allowing food companies up to 12 months to achieve full compliance^(19)^.

In 2019, Argentina issued three new resolutions (1/2018^(20)^, 4/2019^(21)^ and 33/2019^(22)^) to update stringent Na targets in food categories such as farinaceous; meat products and soups and bouillons and to include new Na targets for dressings such as mayonnaise, tomato-based dressing and sauces. In both cases, companies will have 18 months to achieve full compliance.

Regarding the nutritional labelling information provided to the consumer, in Argentina, it is regulated by the Argentine Food Code. This code establishes that information about the quantitative content of Na (mg/portion size) is mandatory on the nutritional information panel on the products^(23)^. The same code also sets the analytical methodology for calculating this critical nutrient^(23)^.

Furthermore, in the Latin American and Caribbean (LAC) region, voluntary regional targets for sodium content were set in 2015 for eleven food categories by the Pan American Health Organisation (PAHO) Technical Advisory Group for Sodium, based on established national targets in the LAC region and endorsed by the Salt Smart Consortium, a group of government and non-governmental organisations and food companies. LAC countries were expected to have met these targets by December 2016^(24)^. In 2021, PAHO updated its Regional Sodium Reduction Targets and established revised targets for 2022 and 2025 for sxiteen food categories and seventy-five subcategories^(2)^.

Previous studies comparing the Na levels of processed foods in Argentina during 2014 and 2018 showed high levels of compliance with the National Law but relevant challenges in specific food categories and with the regional targets^(25,26)^. For example, in the last analysis, almost 50 % of products contained Na levels above the 2015 PAHO lower targets^(26)^. Therefore, in order to know the progress in the implementation of the national sodium policy in Argentina and to compare Na levels in relation to the regional limits recommended by PAHO for Na in foods^(2)^, the objectives of this study were (a) to assess the current Na levels in a variety of processed food groups and categories available in the Argentinean market; (b) to monitor compliance with the maximum levels set forth by Act 26 905 and the most recent amendments and (c) to compare the current Na content levels with the updated regional targets. In this study, we measured progress in implementing the national sodium policy in Argentina and also analyzed Na levels relative to the PAHO regional recommended limits for salt/Na in foods.

## Materials and methods

This was a cross-sectional, systematic survey of pre-packaged food products available in the Argentinian food supply assessing sodium content as reported in nutrition information panels. Sodium levels were compared against the maximum values set in Act 26 905 and in the updated PAHO regional targets. This study was part of the collaborative regional study that included Argentina, Peru, Panama and Costa Rica. The purpose was to compare the Na content of foods among these countries, aiming to understand the extent to which regional targets are being met in each case.

### Data collection

Data were collected during March 2022 in Buenos Aires city in two of the main supermarket chains (JUMBO and Chango Más). The selected stores are among the six leading retailers in Argentina, which altogether represent 80 % of the grocery retail market^(27)^.

Data collection was conducted by members of the research team on-site, with written store management approval. Each product was surveyed using the Food Label Information and Price for Latin American and Caribbean countries smartphone data collector app and web-based software developed by the University of Toronto (U of T)^(28)^. Food Label Information and Price for Latin American and Caribbean is a food composition database software (web and mobile) that provides a shorter and more efficient food collection and data processing approach. Data collection consisted of scanning the bar code of each product and taking photographs of all sides including all products on the shelf, which can encompass both products similar to those included in the previous analysis^(25,26)^ and new products available on the market. This information was then uploaded to the FLIP database for processing and analyses. For each product, the manufacturer, brand and product names, serving size, container size, ingredients and complete nutritional information (where applicable) of the product as sold and as consumed (serving/g or ml) were entered. Data collectors walked along and observed all the aisles and product displays in each store to ensure that all products available for purchase and belonging to the food categories of interest were recorded. Food products sold at more than one retailer were captured only once. Quality assurance measures were implemented to detect duplicate products and ensure accuracy of data entry (e.g. rank ordering data to identify outliers, calculation of Atwater factors) and food category classification.

### Food product categorisation

**Fig. 1:**
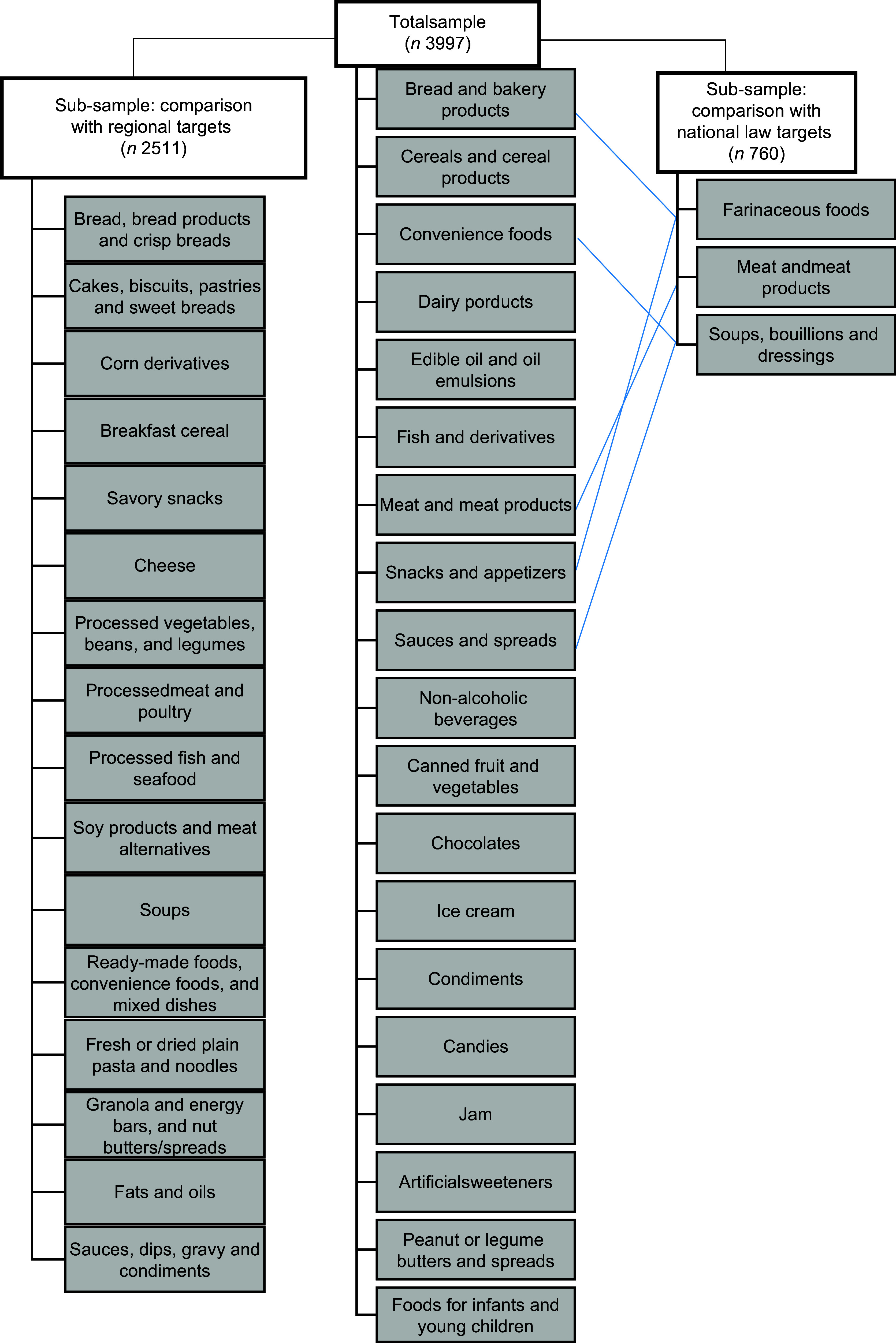
Food groups included in the total sample and their relationships with food groups whose maximum sodium levels are set by National Law and regional targets

Food groups were defined to include products manufactured from the same raw material and with similar nutritional content (e.g. bread and bakery products and dairy products). Food categories included products with the same manufacturing process (e.g. biscuits and bread, within the bread and bakery products groups)^(29)^. The final food categorisation system included nineteen food groups: bread and bakery products; cereals and cereal products; convenience foods; dairy; edible oils and oil emulsions; fish and derivatives; meat and meat products; snacks and appetizsers; sauces and spreads; non-alcoholic beverages; canned fruit and vegetables; chocolates; ice cream; condiments, candies; jam; artificial sweeteners; peanut or legume butters and spreads and foods for infants and young children (Fig. 1).

### Data analysis

For the comparison of the surveyed products against the current Na content targets established by Act 26 905,^(19)^ we matched food groups and categories from our database with the food groups and categories included in the law: farinaceous foods; meat and meat products and soups, bouillons and dressings (Fig. 1). These groups were also classified in thirty-three categories. Additionally, food products were classified based on the updated regional targets, where foods are grouped into sixteen major categories and seventy-five subcategories defined by the PAHO^(2)^. In order to be consistent with food categorisation across countries, as part of the collaborative regional study, food categorisation was validated by the U of T research team, and minor discrepancies were resolved (Fig. 1). Data were analysed and compared against the different Na targets established in the Updated PAHO Regional Sodium Reduction Targets based on mg/100 g for 2022 and 2025^(2)^.

### Statistical analyses

The Na content in foods was obtained from the nutrition facts table (mg/serving) and was converted to standardised units (mg/100 g) considering the products as consumed. Median values were used to characterise the distribution of the data set in each food group and category. The mean and the range are included as a reference; the percentage coefficient of variation (C.V. %) is provided as an alternative index of dispersion. The median Na content of products belonging to food groups/categories considered in the National Law was compared with the Na targets established by the law and the updated regional targets. We present the percentage of products in each category that exceed the Na thresholds in each system. All data analyses were conducted using Python.

## Results

### Sodium content by Argentinean food categories

The total sample (*n* 3997) was mainly composed of the following five categories: bread and bakery products (*n* 691, 17·3 %), dairy products (*n* 560, 14·0 %), cereal and cereal products (*n* 443, 11·1 %), convenience foods (*n* 440, 11·0 %) and non-alcoholic beverages (*n* 360, 9·0 %).

The five categories with the highest median Na content were meat and fish condiments (median: 13 500 mg/100 g), appetisers (median: 1900 mg/100 g), luncheon meat and sausages (median: 921·21 mg/100 g), dressings (median: 777·5 mg/100 g) and leavening flour (median: 757 mg/100 g) (Table 1).

**Table 1:** Sodium content of processed foods in Argentina by category (*n* 3997)

Food group	Food category	Products (*n*)	Products (%)	Mean	Std	CV	Median	Range (min–max)
Bread and bakery products	Bread	140	3·5	345·4	239·3	69·3	310·0	0–2110
	Toast	33	0·8	514·4	254·9	49·6	560·0	0–783·3
	Biscuits	373	9·3	316·8	233·7	73·8	271·4	0–1366·7
	Bakery products	145	3·6	346·1	248·6	71·8	291·7	232·3–2110
Total		691	17·3					
Cereal and cereal products	Cereal bars	33	0·8	137·1	118·5	86·5	104·4	0–428·6
	Breakfast cereal	92	2·3	211·3	183·3	86·7	207·5	0–810
	Pasta and noodles	276	6·9	126·1	201·8	160·1	11	0–765
	Soy-based products	21	0·5	416·3	99·3	23·8	388·8	305·1–784
	Leavening flour	12	0·3	638·3	404	63·3	757	0–1460
	Others	9	0·2	292·1	216·4	74·1	383·5	0·4–494·7
Total		443	11·1					
Convenience foods	Puff pastry for pies	19	0·5	621·6	237·7	38·2	630	203·3–1016·7
	Puff pastry for empanadas	32	0·8	715·4	445·9	62·3	652	208–2901·8
	Soup	39	1·0	295	121·6	41·2	280	32–676·4
	Bouillon cubes	19	0·5	284·5	100·9	35·5	314	16·8–373·2
	Pizza	24	0·6	476·3	276·6	58·1	552·5	89·2–972·5
	Ready-made meals	86	2·2	327·8	145·1	44·3	322·7	14–702·4
	Pre-cooked meals	40	1·0	311·4	189·9	61	385·2	18·8–580·8
	Pre-mixtures	82	2·1	314·6	194·4	61·8	282·1	0–1035
	Instant dessert mixtures	81	2·0	85·2	64·6	75·9	80·8	0–507·5
	Frozen vegetables	18	0·5	65·9	55·1	83·7	53·5	22–226
Total		440	11·0					
Dairy	Cheese	262	6·6	635·6	425·5	66·9	558·3	0–3300
	Dairy-based desserts	47	1·2	90·9	21·4	23·6	91	35–147·4
	Yoghurt	196	4·9	59·3	20·4	34·3	58·5	0–123·8
	Condensed milk	5	0·1	120	8·7	7·2	115	115–135
	Milk cream	15	0·4	38·1	14·2	37·2	41·3	0–56
	Flavored or sweetened milk	35	0·9	67·1	23·7	35·3	63·5	13·5–117·2
Total		560	14·0					
Edible oils and oil emulsions	Butter	13	0·3	71·4	84·6	118·5	22	0–260
	Margarine	7	0·2	465·7	406·6	87·3	240	200–1310
Total		20	0·5					
Fish and derivatives	Canned tuna	23	0·6	363·3	118	32·5	341·7	196·7–680
	Canned mackerel	7	0·2	248·8	113·7	45·7	255	128·3–473·3
	Other fish	19	0·5	925·7	1786·2	193	338·3	0–5913·3
	Canned sardines	1	0·0	576·7			576·7	576·7–576·7
	Breaded fish products	4	0·1	243·9	139·8	57·3	234·6	87–419·2
Total		54	1·4					
Meat and meat products	Hamburger	21	0·5	660·2	112·1	17	666·7	395–801·3
	Luncheon meat and sausages	99	2·5	1246	890·2	71·4	940	300–5460
	Spreads	12	0·3	670·8	253·5	37·8	750	260–970
	Breaded chicken products	27	0·7	487·7	120·6	24·7	467·1	46·2–610
	Others	33	0·8	702·2	233	33·2	643·8	286·2–1405
Total		192	4·8					
Snacks and appetisers	Snacks	164	4·1	660	225·4	34·2	676	25–1292
	Appetisers	48	1·2	1907·3	659·7	34·6	1950	326·2–2885
Total		212	5·3					
Sauces and spreads	Salsas	29	0·7	270·2	232·4	86	288·3	0–1210
	Dressings	167	4·2	988·2	1021·8	103·4	775	0–8060
Total		196	4·9					
Non-alcoholic beverage		360	9·0	17·8	17·9	100·7	12·5	0–96
Canned fruit and vegetables	Canned vegetables	106	2·7	168·7	144·9	85·9	165·3	0–920
	Canned fruit	17	0·4	8	7·8	97·1	5·9	0–25
Total		123	3·1					
Chocolates	Alfajores	64	1·6	108·6	49·9	45·9	109·4	11·4–291·8
	Chocolate icing	10	0·3	104	75·7	72·8	80	40–244
	Dipping chocolate	1	0·0	12			12	12–12
	Chocolate bars	108	2·7	103	73·3	71·2	105·9	0–548
	Cocoa powder	14	0·4	53	27·2	51·2	63·5	0–90
	Others	71	1·8	115·1	81·8	71·1	100	0–470
Total		268	6·7					
Ice cream		82	2·1	45·9	32·2	70	48·7	
Condiments	Meat and fish condiments	15	0·4	11 968·2	5294·2	44·2	13 500	445–17 120
	Bouillon cubes and powders	17	0·4	3609·7	6854·7	189·9	349·2	24–24 000
	Others	6	0·2	2344·4	4666·8	199·1	0	0–11 666·7
Total		38	1·0					
Candies		122	3·1	46·4	67·7	145·8	29·7	0–307·7
Jam	Dulce de leche	29	0·7	155·8	45·7	29·3	150	46·2–225
	Jam	93	2·3	22·8	61·8	270·5	0	0–250
	Others	10	0·3	56·8	89	156·9	0	0–250
Total		132	3·3					
Artificial sweeteners	Artificial sweeteners in powder or tablet	28	0·7	44·6	236·2	529·2	0	0–1250
	Liquid artificial sweeteners	17	0·4	169·4	254·4	150·2	0	0–640
Total		45	1·1					
Peanut or legume butters and spreads		11	0·3	301·2	361·5	120	60	0–800
Foods for infants and young children		8	0·2	59·8	46	76·9	43·9	27·5–169·2
Total		3997	100					

Na content was very variable among products belonging to the same category. Maximum variability was reported for artificial sweeteners in powder or tablet (range: 0–1250·7 mg/100 g, CV: 529·2 %), jam (range: 0–250 mg/100 g. CV: 270·5 %), other condiments (range 0–11 666·7 mg/100 g. CV: 199·1 %), other fish products (range: 0–5913·3 mg/100 g. CV: 193 %) and bouillon cubes and powders (range: 24–24 000 mg/100 g. CV: 189·9 %) (Table 1).

The categories with the highest maximum Na values are bouillon cubes and powders (24 000 mg/100 g), meat and fish condiments (17 120 mg/100 g), other condiments (11 666·7 mg/100 g), dressings (8060 mg/100 g), other fish (5913·3 mg/100 g), luncheon meat and sausages (5460 mg/100 g), cheese (3300 mg/100 g), puff pastry for empanadas (2901·8 mg/100 g), appetisers (2885 mg/100 g) and bread (2110 mg/100 g) (Table 1).

### Comparison of Sodium Content against the Maximum Levels Set by National Act 26 905

There were 760 products in our database (*n* 3997) that were included in the food groups and categories regulated by Act 26 905. Most of them belong to the farinaceous food group (*n* 518), followed by meat and meat products (*n* 135) and soups, bouillons and dressings (*n* 107). Although the observed median values were below the Na targets in all product categories, eighteen of the thirty-three categories considered in this analysis included one or more specific products whose Na levels were above the category target. Of the 760 products analysed, 6·3 % (*n* 48) were found to exceed the Na content targets set by law (Table 2).

**Table 2:** Comparison of the observed sodium content with the maximum levels set forth by act 26 905 (*n* 760)

Food group	Food category	Products (*n*)	Median (mg/100 g)	Maximum levels Act. 26·905 (mg/100 g)	Products above targets	
					*n*	%
Meat and meat products	**Cooked sausages** *	**36**	**921·2**	**1136**	**4**	**11·1**
	**Luncheon meat**	**31**	**827·5**	**1136**	**5**	**16·1**
	Dry sausages	16	1478·7	1805	0	0
	Fresh sausages	4	789	903	0	0
	Hamburgers	21	666·7	808	0	0
	Breaded chicken products	27	467·1	699	0	0
Total meat and meat products		135	–	–	9	6·7
Farinaceous	**Bran crackers**	**16**	**699·9**	**890**	**1**	**6·3**
	**Non-bran crackers**	**36**	**553·3**	**890**	**3**	**8·3**
	Snack crackers	36	824	1340	0	0
	Corn flour snacks	5	900	900	0	0
	Mix snacks	2	361·2	900	0	0
	**Cheese puffs**	**11**	**596**	**900**	**1**	**9·1**
	Cheese-flavored sticks	9	768	900	0	0
	**Potato chips**	**38**	**552**	**900**	**1**	**2·6**
	Salted peanuts	22	604	900	0	0
	Nachos	8	408	900	0	0
	**Other snacks**	**33**	**752**	**900**	**3**	**9·1**
	**Dry sweet cookies**	**118**	**245**	**485**	**1**	**0·8**
	**Filled sweet cookies**	**106**	**198**	**405**	**3**	**2·8**
	**Wholemeal bread**	**29**	**416**	**503**	**6**	**20·7**
	**White bread**	**17**	**448**	**476**	**3**	**17·6**
	**Hot dog buns**	**12**	**435**	**476**	**2**	**16·7**
	**Hamburger buns**	**19**	**432**	**476**	**3**	**15·8**
	Frozen bread	1	500	527	0	0
Total farinaceous		518	–	–	27	5·2
Soups, bouillons and dressings	Bouillons	19	314	405	0	0·0
	**Clear soup**	**13**	**278·4**	**330**	**2**	**15·4**
	Cream soup	8	255·6	290	0	0
	**Instant soup**	**10**	**225·2**	**330**	**1**	**10·0**
	**Mayonnaise**	**27**	**733·3**	**833**	**6**	**22·2**
	**Ketchup**	**13**	**833·3**	**970**	**2**	**15·4**
	Golf sauce	2	758·3	850	0	0
	**Ready-made sauces in sachet**	**14**	**290**	**315**	**1**	**7·1**
	Ready-made canned sauces	1	102·3	315	0	0
Total soups, bouillons and dressings		107	–	–	12	11·1
Total		760			48	6·3

* Categories highlighted in bold indicate those products that exceed the national maximum targets set by the law.

Non-compliances with the law were found in all food groups: soups, bouillons and dressings (11·2 %), meat and meat products (6·7 %) and farinaceous products (5·21 %). Within soups, bouillons and dressings, the categories mayonnaise (22·2 %), ketchup (15·4 %), clear soup (15·4 %), instant soup (10 %) and ready-made sauces in sachet (7·1 %) were found to be in non-compliance with the law. Among meat and meat products, only luncheon meat and cooked sausages were found to be in non-compliance with the law (16·1 % and 11·1 % in non-compliance respectively). Finally, regarding farinaceous group, the categories with most of the non-compliant products were wholemeal bread (20·7 %), white bread (17·6 %), hot dog buns (16·7 %), hamburger buns (15·8 %) and cheese puffs (9·1 %) (Table 2).

### Comparison of current sodium levels in Argentina with regional sodium targets

This analysis was performed on 1449 of the total 3997 surveyed products from eight categories and twenty-seven sub-categories included in the regional targets for 2022 and 2025 considered the most relevant at national level. The eligibility criteria are those subcategories covered by the National Law and subcategories that represent the main sources of Na in the Argentine population diet.

Of these products, 51·1 % (*n* 741) were found to exceed the regional target for Na content (mg/100 g) for 2022 and 57·9 % (*n* 840) for 2025. Products were found in all categories that exceeded the regional targets for 2022 and 2025 (Table 3).

**Table 3:** Comparison of sodium content in categories and sub-categories included in the act 26 905 and the main sources of sodium in Argentina with the 2022 and 2025 PAHO sodium regional targets (*n* 1449)

PAHO sodium major category	PAHO sodium sub category	Total products (*n*)	PAHO 2022 sodium targets (mg/100 g)	Products above PAHO 2022 sodium targets		PAHO 2025 sodium targets (mg/100 g)	Products above PAHO 2025 sodium targets	
				*n*	%		*n*	%
Bread, bread products and crisp breads	Pantry and hearth bread, rolls and buns	102	340	90	88·2	280	91	89·2
	Other bread products	118	400	75	63·6	350	78	66·1
Cakes, biscuits, pastries and sweet breads	Savory biscuits and crackers	144	640	74	51·4	580	82	56·9
	Cookies and sweet biscuits	342	225	132	38·6	200	148	43·3
Savory snacks	Nuts, seeds, and kernels, seasoned and candied	36	265	34	94·4	220	34	94·4
	Chips, popcorn, and/or extruded snacks	101	530	73	72·3	470	78	77·2
	Pretzels and snack mixes	3	800	2	66·7	670	2	66·7
	Other savory snacks	3	525	1	33·3	430	1	33·3
Cheese	Fresh cheese (i.e. fresh mozzarella and others)	62	480	32	51·6	400	35	56·5
	Soft cheese (i.e. unripened goat cheese and cream cheese)	59	420	15	25·4	380	17	28·8
	Semi-hard cheese (mozzarella, cheddar and others)	64	650	21	32·8	590	26	40·6
	Hard cheese, grated and ungrated	52	1300	5	9·6	1200	5	9·6
	Processed cheese	19	1000	7	36·8	900	8	42·1
Processed meat and poultry	Packaged deli meats – fully cooked	41	900	14	34·1	800	25	61
	Packaged dry-cured deli meats – dry cured, fermented, no thermal process	21	1350	15	71·4	1200	17	81
	Sausages – uncooked	6	600	5	83·3	500	6	100
	Sausages – cooked	16	840	10	62·5	770	10	62·5
	Uncooked bacon – belly	1	700	1	100	590	1	100·0
	Burgers, meatballs, meatloaf and breaded meat and poultry	66	540	39	59·1	500	48	72·7
	Ham, canned meat and poultry, and uncooked, pickled, cured and smoked meats that are not deli meats	14	915	8	57·1	790	9	64·3
	Patés and meat spreads	13	720	11	84·6	600	11	84·6
Soups	Wet and dry soups (as consumed)	18	260	7	38·9	230	11	61·1
	Noodles in broth (as consumed)	27	330	14	51·9	275	24	88·9
Fats and oils	Mayonnaise	31	670	23	74·2	600	29	93·5
Sauces, dips, gravy and condiments	Bouillon cubes and powders (as sold)	35	18 000	14	40	16 000	23	65·7
	Tomato sauce	26	300	4	15·4	240	4	15·4
	Ketchup and similar tomato type condiments (as consumed)	29	800	15	20·2	780	17	27·2
Total		1449		741	51·1		840	57·9

**Fig. 2:**
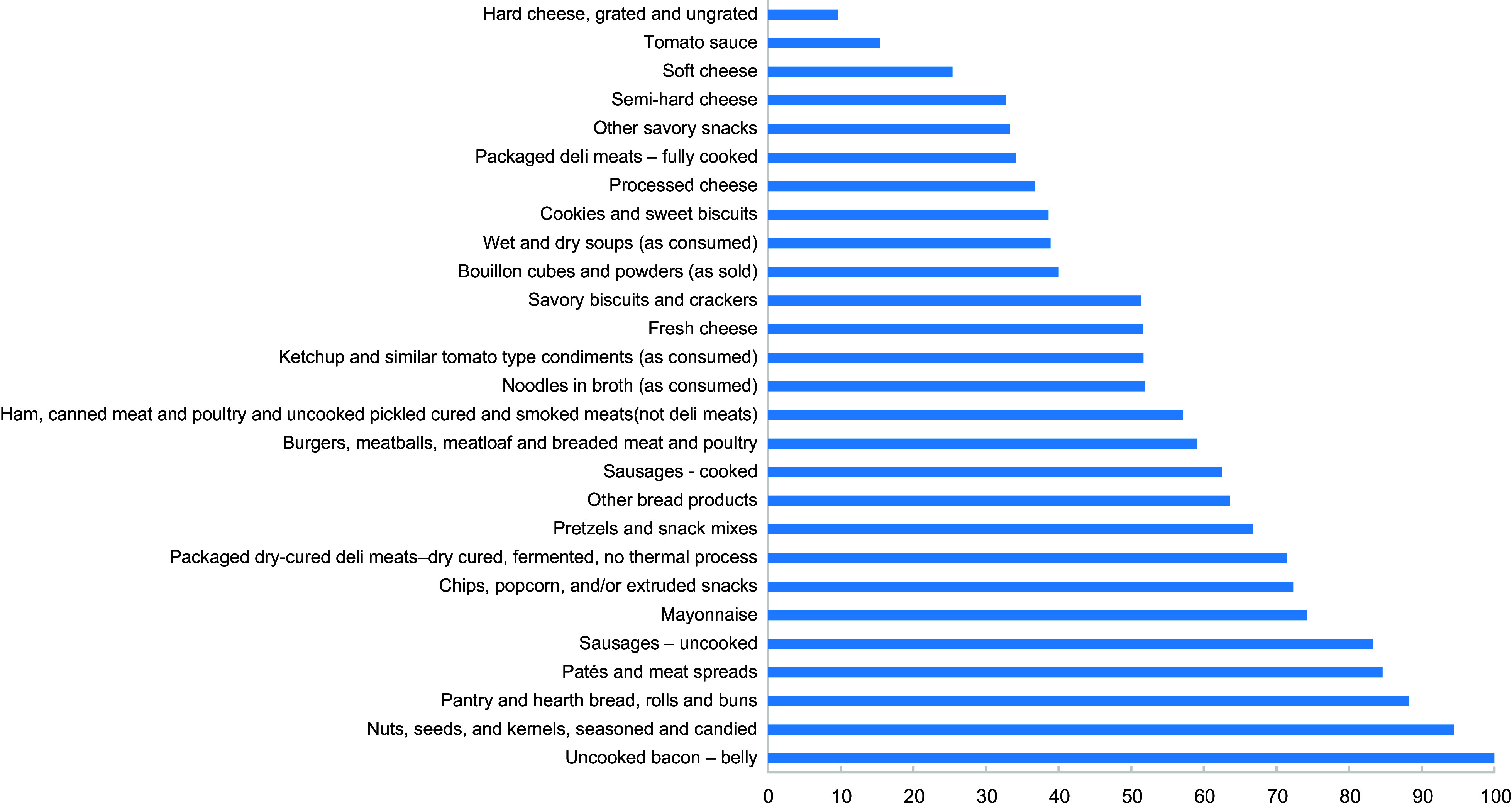
Proportion of products exceeding the PAHO 2022 Sodium Reduction Targets

**Fig. 3:**
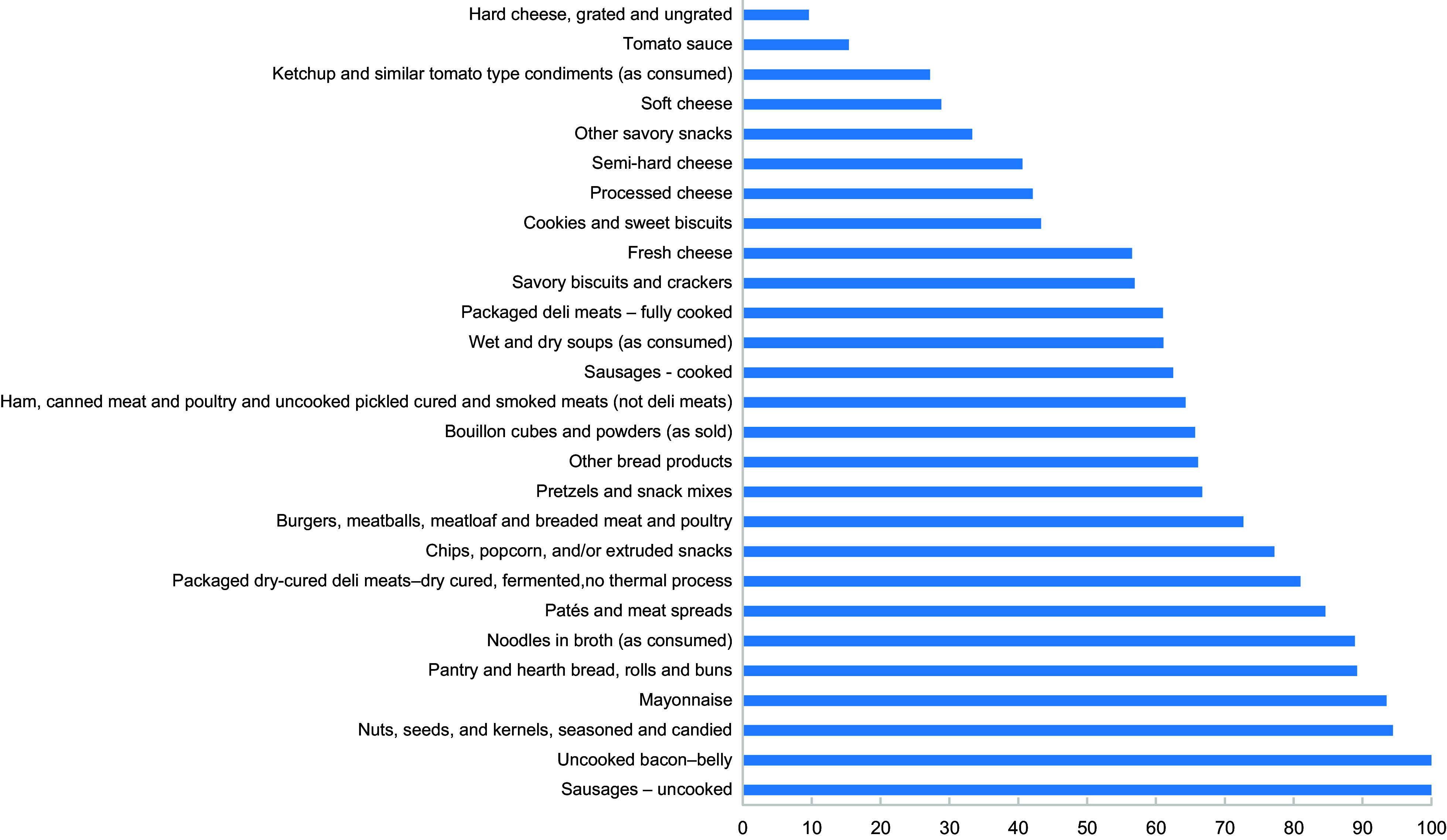
Proportion of products exceeding the PAHO 2025 Sodium Reduction Targets

We observed that the highest percentage of products that exceeded the regional targets for 2022 were the following: uncooked bacon – belly (*n* 1, 100 %), nuts, seeds, and kernels, seasoned and candied (*n* 34, 94·4 %), pantry and hearth bread, rolls and buns (*n* 90, 88·2 %), patés and meat spreads (*n* 11, 84·6 %), sausages – uncooked (*n* 5, 83·3 %), mayonnaise (*n* 23, 74·2 %), chips, popcorn, and/or extruded snacks (*n* 73, 72·3 %), packaged dry-cured deli meats – dry cured, fermented, no thermal process (*n* 15, 71·4 %), pretzels and snack mixes (*n* 2, 66·7 %) and other bread products (*n* 75, 63·6 %) (Table 3 and Fig. 2). The main sub-categories that exceeding the 2025 regional targets were uncooked bacon – belly (*n* 1, 100 %), sausages – uncooked (*n* 6, 100 %), nuts, seeds, and kernels, seasoned and candied (*n* 34, 94·4 %), mayonnaise (*n* 29, 93·5 %), pantry and hearth bread, rolls and buns (*n* 91, 89·2 %), noodles in broth (*n* 24, 88·9 %), pates and meat spreads (*n* 11, 84·6 %), packaged dry-cured deli meats – dry cured, fermented, no thermal process (*n* 17, 81 %), chips, popcorn, and/or extruded snacks (*n* 78, 77·2 %) and burgers, meatballs, meatloaf and breaded meat and poultry (*n* 48, 72·7 %) (Table 3 and Fig. 3).

Table S1 shows the analysis comparing total regional targets for 2022 and 2025 including fifteen categories and sixty-three sub-categories. The other twelve categories were excluded because no matches were found with PAHO categories.

## Discussion

This study is the third analysis of Na content in processed foods as reported in the nutrition facts table performed in Argentina. It is part of an ongoing effort to independently monitor the implementation of the National Law. Previous analyses were performed in 2014^(25)^ and 2018^(26)^ with the same methodology.

Although Argentina was one of the pioneering countries in the LAC region in promoting mandatory policies to reduce Na intake and while the law was updated in 2019 to incorporate stricter maximum limits as in the case of soups and include new categories such as mayonnaise and ketchup, the results of this study demonstrate there is still considerable room for improvement. In the first place, the current study identifies a high level of compliance with the national Na targets and a significant variability in the Na content within the same category, which has certain implications that are noteworthy in the context of current policy. Likewise, the finding that more than 50 % of the products exceed regional targets, both with respect to the limits for 2022 and 2025, poses challenges that deserve highlighting.

First and foremost, the present study demonstrated that the existing limits are too lax and need further adjustment. The results indicate that the majority of products covered by national regulations already meet the established maximum Na levels. This finding is consistent with previous local evidence^(26)^, and monitoring evaluations conducted by the Argentinean Ministry of Health^(30,31)^. Simultaneously, the significant variability in Na content within each category suggests that it is possible to reduce the levels of this critical nutrient without compromising the organoleptic characteristics of the product. This aligns with previous analyses conducted in Argentina^(25,26)^ and in other countries that have observed high variability in Na content within each category, especially in categories such as processed meats and fish^(32,33)^.

Second, it is important to update the law to include new targets for those food categories with the highest Na content and that represent the main Na dietary source. For example, meat and fish condiments, leavening flour and appetisers, which were identified among the categories with the highest Na content and that are not yet included in the Argentinean law require the application of maximum limits. Furthermore, it is important to consider that a wider range of processed and packaged food categories that contribute significantly to high Na diets, such as cheese and puff pastry,^(34)^ and that were also identified in this study among the categories with the highest maximum values of the nutrient, should be added.

Third, the findings of the current study underscore the urgent necessity to advocate for a comprehensive and systematic surveillance approach aimed at fortifying the monitoring and enforcement system. Both this study and prior local research^(25,26)^ consistently reveal that products falling into categories such as bread and processed meats are in violation of the law and have been identified as categories with the extreme upper limits of sodium content. The latest state monitoring reports from the year 2019 also highlight violations within the snack category^(31)^. This underscores the importance of implementing, at a minimum, annual monitoring by the government across all food categories covered by the law, extending to the monitoring of breads sold directly in bakeries.

Finally, a key conclusion drawn from the present research highlights the imperative need to revise the National Sodium Reduction Law in alignment with regional public health standards. The results reveal that, when comparing the Na content of products against regional targets, more than 51 % exceed the regional target for sodium content (mg/100 g) for 2022, and this proportion increases to over 57 % for 2025. This stark contrast is largely attributable to the fact that regional targets encompass a broader range of categories than the National Law. Furthermore, even within categories already addressed by the National Law, such as processed meats or savory snacks, the PAHO targets impose more stringent limits. It is worth noting that regional targets were established based on the Na levels in products within each food category across various countries in Latin America and the Caribbean. These targets aim to achieve reductions of 15 % and 30 % by 2022 and 2025, respectively, contributing to the global objective of reducing Na consumption in the population^(35)^. Also, at a global level, WHO published in 2021 Sodium benchmarks for different food categories that complement the national and regional efforts and tend to serve as a global standard for the monitoring of Na content in food products^(36)^. Although to date there have been no studies demonstrating the potential dietary and health impact resulting from the adoption of PAHO’s regional targets, in Australia, it has been estimated that meeting the WHO’s sodium benchmarks for packaged foods could lead to a decrease of 404 mg/d in the average Na intake among adults, equivalent to a 12 % reduction^(37)^. Furthermore, in Canada, a simulation modeling study estimated that fully meeting current voluntary sodium reduction targets in processed foods could result in a reduction of 459 mg/d in Na intake, representing an approximate 17 % decrease. This reduction could potentially avert or delay 2176 (95 % UI 869–3687) deaths from CVD^(38)^.

It is noteworthy to mention that Argentina has enacted a comprehensive law, the ‘Promotion of Healthy Food Law,’ aimed at fostering healthy food environments^(39)^. This legislation, fully implemented in November 2023, complements ongoing national initiatives to reduce sodium intake in Argentinean food products. The law incorporates front-of-pack labeling, enabling consumers to easily and accurately identify products containing excessive amounts of critical nutrients such as Na, sugar or fat. The thresholds for these labels are established by the Pan American Health Organization Nutrient profile model, which is based on the WHO Population Nutrient Intake Goals^(40)^. Furthermore, the law encourages the food and beverage industry to lower Na levels in pre-packaged foods to avoid triggering Na warning labels. A similar regulatory approach implemented in Chile demonstrated a positive impact on the reformulation of processed foods, leading to a significant reduction in Na content in groups such as fats and oils, spices, condiments and sauces^(41)^. Evaluations also indicated a noteworthy decrease in the proportion of products labelled as ‘high in’ Na, from 74 % to 27 %, in categories including savory spreads, cheeses, ready-to-eat meals, soups, and sausages^(42)^. These findings illustrate the feasibility of reducing Na levels in crucial food categories. Ultimately, it is crucial that both policies, which are part of the SHAKE package,^(43)^ are effectively implemented and monitored to prevent processed foods from becoming a major source of Na in Argentina, where the consumption of these manufactured foods is rapidly increasing^(44–46)^.

This study presents certain limitations such as the fact that the Na content used for these analyses relied on food labelling values, without chemical analysis verification. The food Argentinian Food Code allows a 20 % difference between food label and laboratory value. Another limitation is that the sample is not representative of all products available in the Argentinean market, such as those sold in informal markets and bakery products; also, inter-store or regional variations in product availability were not addressed because of the small number and the location of the stores surveyed – Buenos Aires city; however, the selected stores are among the main six supermarket chains in Argentina, that altogether represent 80 % of the grocery retail market^(27)^.

The main strength of this study is the use of a continuous standardised methodology for examining Na content of foods that allows for ongoing monitoring over time and worldwide comparisons. Furthermore, this analysis serves as a valuable instrument for autonomously overseeing the existing national policy and pinpointing areas of strength and areas that could be enhanced. The insights gained from this monitoring process can also streamline the implementation of Na reduction initiatives in other countries across the Americas.

### Conclusions

To conclude, this study presents valuable input to improve the National Law in line with regional targets developed by PAHO. Results demonstrate that a large proportion of food products are already meeting the national Na reduction targets and the feasibility for further reductions. To achieve meaningful reductions in Na consumption, it is necessary to update the national Na reduction targets and to include target-setting across a broader range of food categories and subcategories. Also, this policy should be part of a comprehensive strategy to be implemented simultaneously in line with the SHAKE package recommended by the WHO to accelerate progress towards reduction of mean population intake of Na in Argentina^(43)^.
